# Low tube voltage CT for improved detection of pancreatic cancer: detection threshold for small, simulated lesions

**DOI:** 10.1186/1471-2342-12-20

**Published:** 2012-07-24

**Authors:** Jon Holm, Louiza Loizou, Nils Albiin, Nikolaos Kartalis, Bertil Leidner, Anders Sundin

**Affiliations:** 1 Division of Medical Physics, Karolinska University Hospital, Huddinge, Stockholm 14186, Sweden; 2 Department of Clinical Science, Intervention and Technology (CLINTEC), Karolinska Institutet, 17177 Stockholm, Sweden; 3 Department of Radiology, Karolinska University Hospital, Huddinge, 14186 Stockholm, Sweden; 4 Department of Radiology, Karolinska University Hospital, Solna, 17176 Stockholm, Sweden; 5 Department of Molecular Medicine and Surgery, Karolinska Institutet, Stockholm 17176, Sweden

**Keywords:** Pancreatic adenocarcinoma, CT, Low tube voltage, Phantom

## Abstract

**Background:**

Pancreatic ductal adenocarcinoma is associated with dismal prognosis. The detection of small pancreatic tumors which are still resectable is still a challenging problem.

The aim of this study was to investigate the effect of decreasing the tube voltage from 120 to 80 kV on the detection of pancreatic tumors.

**Methods:**

Three scanning protocols was used; one using the standard tube voltage (120 kV) and current (160 mA) and two using 80 kV but with different tube currents (500 and 675 mA) to achieve equivalent dose (15 mGy) and noise (15 HU) as that of the standard protocol.

Tumors were simulated into collected CT phantom images. The attenuation in normal parenchyma at 120 kV was set at 130 HU, as measured previously in clinical examinations, and the tumor attenuation was assumed to differ 20 HU and was set at 110HU. By scanning and measuring of iodine solution with different concentrations the corresponding tumor and parenchyma attenuation at 80 kV was found to be 185 and 219 HU, respectively.

To objectively evaluate the differences between the three protocols, a multi-reader multi-case receiver operating characteristic study was conducted, using three readers and 100 cases, each containing 0–3 lesions.

**Results:**

The highest reader averaged figure-of-merit (FOM) was achieved for 80 kV and 675 mA (FOM = 0,850), and the lowest for 120 kV (FOM = 0,709). There was a significant difference between the three protocols (p < 0,0001), when making an analysis of variance (ANOVA). Post-hoc analysis (students *t*-test) shows that there was a significant difference between 120 and 80 kV, but not between the two levels of tube currents at 80 kV.

**Conclusion:**

We conclude that when decreasing the tube voltage there is a significant improvement in tumor conspicuity.

## Background

Pancreatic ductal adenocarcinoma (PDAC) is associated with a dismal prognosis. The overall 5 year survival rate is less than 5% and even after potentially curative surgery this increases to only 20% [1]. Tumor size is an important prognostic factor and increasing size correlates with a higher rate of unresectable tumors and decreased survival [2]. For this reason it is important to detect pancreatic tumors while they are small and still resectable. Technological advances in multi-detector computed tomography (MDCT) combined with its wide availability have made it the modality of choice for diagnosing and staging pancreatic malignancies [3]. MDCT is highly sensitive in detecting large tumors: 100% sensitivity for tumors > 2 cm [4,5] but for small tumors, <2 cm, sensitivity is lower (60-77%) [4,5]. Recent studies have shown that MDCT and Magnetic Resonance Imaging (MRI) have comparable diagnostic accuracy [6,7] with MRI probably offering an advantage for liver metastases [7]. Our clinical impression, using a 64-channel MDCT and a triple-phased protocol, is that the sensitivity to detect 1–2 cm tumors is higher than is stated in the literature but for very small tumors, <1 cm, the detection rate is very low and needs to be improved.

Imaging pancreatic cancer with MDCT needs contrast enhancement in at least two phases, the pancreatic parenchymal phase (PPP) and the portal venous phase (PVP). In the obligate PPP the normal pancreatic parenchyma enhances avidly whereas the vast majority of pancreatic adenocarcinomas (PDAC) are hypointense, due to the high fibrous tissue content of the tumor [8]. The key to the PDAC diagnosis is to achieve as high an attenuation difference as possible between the normal pancreatic parenchyma and the tumor [9]. Reducing the tube voltage can increase this contrast between tumor and normal parenchyma [10]. The main disadvantage of low tube voltage CT is the increased image noise, which until recently has been difficult to overcome because of limitations in the output of the x-ray tubes. The increased contrast is achieved by an increased photoelectric effect and a decreased Compton scattering, resulting in a higher attenuation of iodinated contrast media [10]. This principle has been used to reduce the radiation dose for CT of the thorax [11,12] and heart [13,14], in patients with low body mass index (BMI) and in children. In recent years, the technique has also been used to improve CT angiography of the pulmonary arteries [15-17] and to facilitate detection of hypervascular liver lesions [18]. In a recent dual-energy MDCT study, the low tube voltage technique improved the enhancement of the pancreas and peripancreatic vasculature in order to improve tumor conspicuity [19].

In this phantom study, the purpose was to investigate whether a decrease of the tube voltage from 120 kVp to 80 kVp could improve the detection of small, low attenuating, solid pancreatic tumors. We decided to assess this in an experimental model whereby small hypoattenuating simulated tumor lesions were mathematically created in a phantom.

## Methods

### Image acquisition

**Figure 1:**
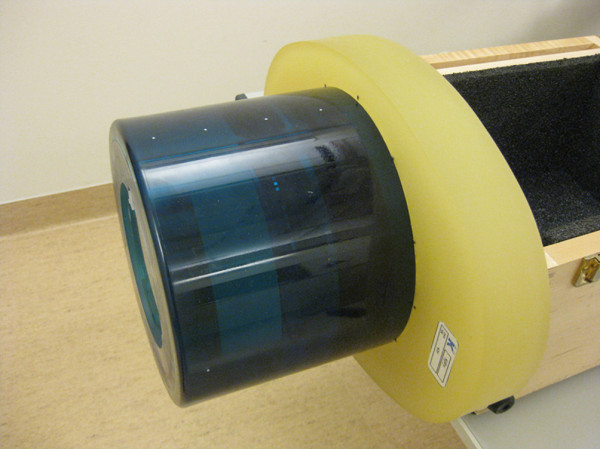
**Image of the phantom.** Image of the Catphan 600 phantom with the body anulus (CTP579) mounted on top of the image uniformity module (CTP486).

A phantom (Catphan® 600, The Phantom Laboratory, Salem, USA) was scanned with a 64-channel MDCT scanner (LightSpeed VCT, GE Healthcare, Milwaukee, USA), using three protocols A, B and C (Table 1). The phantom consisted of five separate modules with different properties. The CTP486 image uniformity module of the Catphan phantom was scanned to acquire uniform images. A body annulus, CTP579, was mounted onto the phantom to better simulate the size of the human trunk (Figure 1).

**Table 1:** Scanning parameters

**Protocol**	**Tube voltage [kVp]**	**Tube Current [mA]**	**CTDIvol [mGy]**	**Noise [HU]**
A. 120 kVp	120	160	15	15
B. 80 kVp	80	500	15	17
C. 80 kVp	80	675	20	15

Protocol A utilizes a tube voltage of 120 kVp, which is the tube voltage in clinical use for pancreatic MDCT in our department. In the low voltage protocols B and C, the tube voltage was decreased to 80 kVp. The tube current in protocol B was increased from 160 mA to 500 mA to achieve the same mean radiation dose as in protocol A (15 mGy volume computed tomography dose index (CTDIvol)). In protocol C, the tube current was increased to 675 mA, resulting in a higher radiation dose of 20 mGy CTDIvol, to attain image noise comparable to that in protocol A (15 HU).

For all three protocols, the X-ray tube rotation time (0.6 seconds), detector configuration (64 x 0.625) and helical pitch factor (0.516) were kept constant. The acquired images were reconstructed using the soft reconstruction algorithm, a display field-of-view (DFOV) of 25 cm, a slice thickness of 3 mm and with an interval of 1.5 mm.

### Creation of cases and lesions

One hundred cases were created and used for each protocol. Twenty-one images were acquired for each case with the method described above. In 57 of the 100 cases we inserted 1 to 3 simulated lesions in random positions.

**Figure 2:**
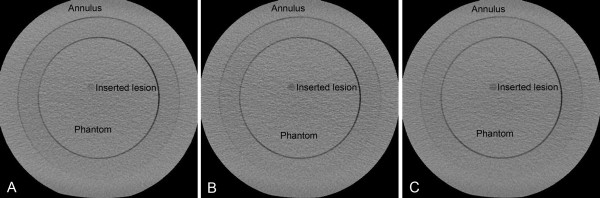
**(A-C) Image of the phantom scanned with protocol A, B and C.** The image includes an inserted lesion with 10 mm diameter and a contrast resulting from 120 kVp scanning (**A**), 80 kVp and 500 mA (**B**) and 80 kVp and 675 mA (**C**). The lesion has a random position inside the inner circle.

The lesions were created assuming a spherical shape. They were calculated by pixel-wise integration of a hemisphere, which is mathematically expressed by:

(1) $$\iint f(x,y)dxdy=\iint \sqrt{r^{2}-x^{2}-y^{2}dxdy}$$

where r is the radius of the lesion and x and y are the Cartesian coordinates in the axial plane of the CT scanner. The integration limits in the xy plane were derived from DFOV information and the size of the image matrix (512 x 512), and are in increments of 0.5 mm in both x and y directions. The integration in z direction is given from the slice thickness and its different positions. Due to the symmetry, integrations were only carried out in one of the quadrants of the hemisphere. The integrations were performed numerically using Mathematica software (Wolfram Research, Champaign, USA). Lesions with a diameter of 2, 3, 4, 5, 6, 8 and 10 mm were created (Figure 2). Because of the 3 mm slice thickness, many of the pixels in the smaller lesions did not only represent the attenuation of the lesion itself, but also the attenuation of the background phantom material, causing partial volume effect problems. Since the lesions had an attenuation close to that of the surrounding phantom material, the CT number for these pixels was computed by assuming a linear combination of the μ values according to their volumetric proportions [20].

### Determination of parenchymal and lesion attenuation

After determining the proportion of lesion and surrounding phantom material in the voxels as described in the previous section, the actual attenuation of the lesion and parenchyma was measured and calculated for both tube voltages. Previous measurements from 15 clinical CT examinations of the pancreas with our standard protocol (120 kVp and 0.75 g I/kg body weight) yielded an attenuation of the normal pancreatic parenchyma of approximately 130 HU in the PPP. These measurements had been performed in examinations reconstructed in 0.625 mm slices, to avoid the effect of partial volume averaging, and by using circular regions of interest (ROIs). The lesions that are usually missed have an attenuation very similar to that of the normal pancreas. Therefore we assumed that the simulated lesions’ attenuation differs from the background by only 20 HU at 120 kVp.

**Figure 3:**
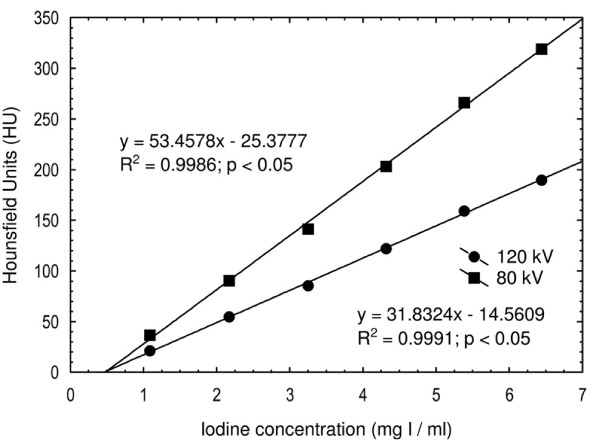
**The mean attenuation.** The mean attenuation (Hounsfield units) for the six test tubes containing different iodine concentrations, positioned in the center position of an RMI phantom and scanned at 120 kV and 80 kV.

Iodine contrast medium (Iomeron 400 mg I/ml) was diluted with water in six different concentrations (1.1, 2.2, 3.2, 4.3, 5.4 and 6.5 mg I/ml) in standard 10 ml plastic vials. These were inserted into the center position of a homogenous phantom (RMI Model 461A, Gammex/RMI, Middleton, USA), which was scanned at 120 kVp and 80 kVp. The attenuation values were plotted against the iodine concentration and correlated linearly (Figure 3). By using this information about attenuation at both tube voltages, all pixel values were calculated based on the assumptions detailed in the previous section.

### Insertion of simulated lesions

The simulated lesions were inserted into the CT images by subtracting the lesion pixel matrix from the uniform phantom image matrix. The phantom background was also adjusted by matrix addition to achieve the same attenuation as the parenchyma. All matrix operations were carried out using Matlab (The Mathworks, Natick, USA). The lesions were randomly positioned in both xy and z planes using the random number generator in Matlab.

### Radiation dose and image noise

**Figure 4:**
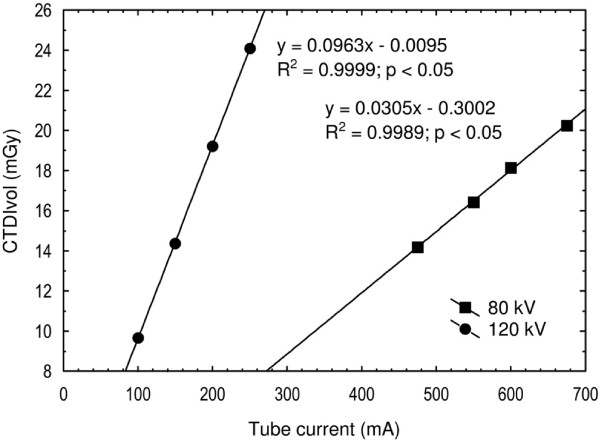
**The radiation dose.** The radiation dose measured as the CTDIvol at 80 kV and 120 kV is shown for different tube currents. From the regression equations one can see that the tube current must be increased from 160 mA to 500 mA when changing the tube voltage from 120 kV to 80 kV to attain the same CTDIvol of 15 mGy.

The CTDI_100_ were measured by using a pencil ionization chamber (DCT10, Wellhöfer, Germany). The measurements were performed with the chamber inside the central (CTDI_100,c_) and the four peripheral holes (CTDI_100,p_) of a 32 cm standard polymethylmethacrylate (PMMA) body phantom. Scans were made for both tube voltages at four different tube currents (Figure 4). Calculations for CTDI_vol_ were performed by using the two well-known equations [21]:

(2) $$CTDI_{w}=\frac{1}{3}CTDI_{100,c}+\frac{2}{3}CTDI_{100,p}$$

(3) $$CTDI_{\mathrm{vol}}=\frac{CTDI_{w}}{pitch}$$

**Figure 5:**
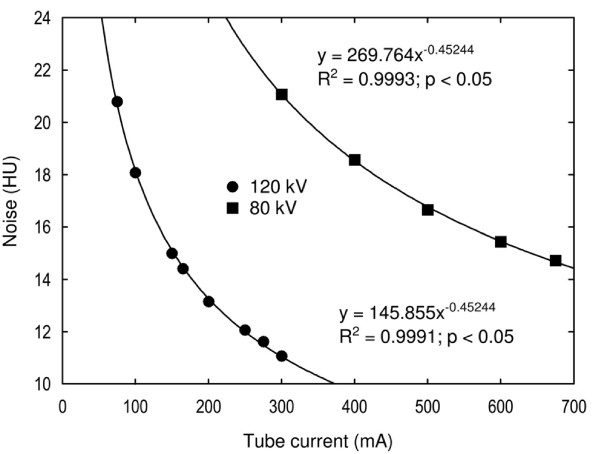
**The image noise.** The image noise at 80 kV and 120 kV is shown for different tube currents. From the regression equations one can see that the tube current must be increased from 160 mA to 675 mA when changing the tube voltage from 120 kV to 80 kV to attain the same image noise of 15 HU.

Image noise was measured in the phantom’s image uniformity module. The phantom was scanned with both tube voltages at different tube currents (Figure 5). For each scan, a circular region of interest (ROI) was used with its size adjusted to cover the whole inner part of the phantom. The standard deviation of the pixels inside each ROI was registered as the amount of the image noise.

Both the radiation dose and the image noise were plotted against the tube current and correlated using appropriate functions (Figures 4 and 5). Regression equations were used to calculate the tube currents for protocols B and C.

### Viewer performance

A standard Picture Archiving and Communicating System (PACS) workstation (Sectra, Linköping, Sweden) was used, with either a one mega pixel color monitor (RadiForce R12, EIZO Nanao Corporation, Ishikawa, Japan) or a three mega pixel grey scale monitor (RadiForce G31, EIZO Nanao Corporation, Ishikawa, Japan). The monitors were calibrated according to DICOM part 14 using a dedicated quality control tool (RadiCS, EIZO Nanao Corporation, Ishikawa, Japan). The images were scaled to fit the monitor, and the radiologists were not allowed to use the zoom tool. The window center was set at the attenuation of the pancreatic parenchyma, which was 130 HU for the 120 kVp images and 217 HU for the 80 kVp examinations. The window width was set to 400 HU for all protocols. Viewing time was unrestricted.

Three radiologists with 24, 21 and 7 years of experience in CT imaging participated in the study. For each scanning protocol, the radiologists were instructed to independently read the 100 cases. They were blinded to CT scanning parameters and to lesion characteristics. They were instructed to indicate suspicious lesions with an arrow marker and rate their level of confidence in the detection of each lesion according to an arbitrary scale ranging from 1 to 4, where 4 indicates the highest confidence and 1 the lowest (Table 2).

**Table 2:** Confidence levels used for rating suspected lesions. The highest rating per case was used as the ROC rating

**Rating**	**Confidence level**
0	Definitely no lesion (no marking)
1	Probably not a lesion
2	Possibly a lesion
3	Probably a lesion
4	Definitely a lesion

Each protocol comprised the same 100 cases, arranged randomly to minimize the memory effect. To familiarize the radiologists to the task, a training session was conducted where they were presented with 10 cases in which the lesions were marked with an arrow. Examples of typical artifacts present in the images were also indicated.

The readers were asked to mark and grade the lesions in two different reading sessions. In the first viewing session they were not allowed to adjust the window setting. In the second viewing session all readers were asked to reinterpret all images, the order of which had been rearranged to minimize the memory effect, but now the readers were instructed that they were free to adjust the window setting according to their own preferences.

### Statistical analysis

The study was analyzed using the receiver operating characteristic method (ROC). The highest rated mark per case was used for the ROC evaluation and the rest of the information was used for descriptive statistics. The collected ROC data were statistically analyzed using DBM-MRMC software version 2.2 [22]. The software first calculated a figure-of-merit (FOM) for each reader and protocol. This was performed by summing the number of ratings for each level of confidence for every actually negative and actually positive case. The false positive fraction (FPF) and the true positive fraction (TPF) were calculated for all possible cut points. A cut point was defined as the point where, as above, the readers’ interpretation is considered as a true positive for an actual positive (tumor case) or a false positive for an actual negative (non-tumor case) [23]. The TPF was then plotted against the FPF and the points were correlated (trapezoidally) and extrapolated to the point of (1,1). The area under the curve (AUC) was the FOM and could be interpreted as the probability that a randomly chosen, actually positive case was rated higher than a randomly chosen, actually negative case [24].

The software performed an analysis of variance (ANOVA) on the FOMs to test any difference between the various scanning protocols, as well as a following post-hoc analysis (*t*-test) to determine exactly where the differences were. Because we had 100 cases but only 3 readers, the analysis was performed by treating the cases as random samples and the readers as fixed samples.

Because the ROC methodology cannot handle information about the number of lesions per case and their localization, the lesion localization fraction (LLF) was calculated. A lesion localization (LL) was defined as a mark that was located not more than 1 cm from a lesion, and the LLF is the LL divided by the total number of lesions. The corresponding non-lesion localization fraction (NLF) was also calculated. A non-lesion localization (NL) was defined as a mark that was located more than 1 cm from a lesion, and the NLF is the NL divided by the total number of cases.

## Results

### Determination of parenchymal and lesion attenuation

The attenuation values for the lesions and the parenchyma at 120 kVp had previously been determined in clinical examinations as 110 HU and 130 HU respectively, and were therefore used for the simulated lesions and parenchyma at 120 kVp. The corresponding attenuation values for the simulated lesions and the simulated normal parenchyma scanned at 80 kVp were 183 HU and 217 HU respectively. Thus, the attenuation difference increased from 20 HU to 34 HU at 80 kVp as compared to 120 kVp.

### Radiation dose and image noise

When the tube voltage was decreased from 120 kVp to 80 kVp, the tube current had to be increased to 500 mA to achieve an equivalent absorbed radiation dose and adjusted up to 675 mA to achieve similar image noise. The CTDIvol for protocols A and B were 15 mGy and for protocol C 20 mGy.

### Viewer performance

**Figure 6:**
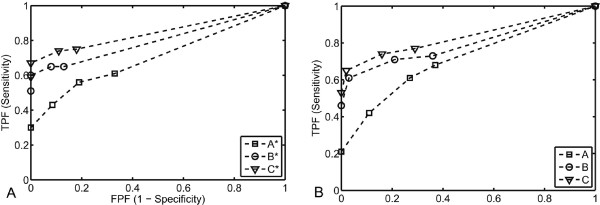
**(A-B) ROC curves with fixed and free-choice window setting.** ROC curves for each scanning protocol interpreted with the fixed window setting (**A**) and free-choice setting (**B**). The FPF is plotted against the TPF for all possible cut points and correlated trapezoidally.

The FOMs for each reader and scanning protocol, determined from the areas under the ROC curves (Figure 6), are presented in Table 3. The highest reader-averaged FOM was acquired for protocol C using a free-choice window setting. The lowest reader-averaged FOM was acquired for protocol A, using a free-choice window setting. The reader-averaged FOM for each protocol, with range in paranthesis as a measure of the interobserver variablility, were as follows: A: 0.713 (0.679-0.741), B: 0.803 (0.785-0.829), C: 0.837 (0.834-0.840), A*: 0.709 (0.706-0.716), B*: 0.807 (0.771-0.842) and C*:0.850 (0.833-0.876). The reader-averaged FOMs differed significantly (p < 0.0001), which in this analysis means that at least two, but not necessarily all, protocols differ. Post-hoc analysis showed better lesion detection when the tube voltage was decreased from 120 to 80 kVp but not when the tube current was increased from 500 to 675 mA at 80 kVp (Table 4). Similar results were achieved by using the predefined fixed window setting and a free-choice window setting. The TPF and FPF for all possible cut points and protocols are presented in table 5.

**Table 3:** FOMs for each reader and scanning protocol together with a reader-averaged FOM. * Indicates a free-choice window setting

**Reader**						
						
	**A. 120 kVp**	**B. 80 kVp**	**C. 80 kVp**	A*. 120 kVp	B*. 80 kVp	C*. 80 kVp
1	0.720	0.785	0.840	0.706	0.771	0.833
2	0.741	0.795	0.836	0.704	0.807	0.842
3	0.679	0.829	0.834	0.716	0.842	0.876
Average	0.713	0.803	0.837	0.709	0.807	0.850

**Table 4:** Inter-protocol comparisons with the highest differences between the FOMs and the lowest p-values at the top

**Protocol comparison**	**Δ FOM**	**P-value**
C* - A*	0.1419	<0.0001
C* - A	0.1372	<0.0001
C - A*	0.1283	<0.0001
C - A	0.1237	<0.0001
B* - A*	0.0979	0.0003
B - A*	0.0943	0.0005
B* - A	0.0933	0.0006
B - A	0.0897	0.0010
C* - B	0.0475	0.0797
C* - B*	0.0439	0.1053
C - B	0.0340	0.2097
C - B*	0.0304	0.2621
C* - C	0.0135	0.6174
A - A*	0.0046	0.8644
B* - B	0.0036	0.8941

**Table 5:** TPF (sensitivity) and The FPF (1 – specificity) for all possible cut points and protocols

**Cut point**	**A. 120 kVp**		**B. 80 kVp**		**C. 80 kVp**	
	**TPF (1-FNF)**	**FPF (1-TNF)**	**TPF (1-FNF)**	**FPF (1-TNF)**	**TPF (1-FNF)**	**FPF (1-TNF)**
0-1	0.678	0.372	0.731	0.357	0.766	0.287
1-2	0.608	0.271	0.708	0.209	0.743	0.163
2-3	0.421	0.109	0.614	0.031	0.655	0.016
3-4	0.211	0.000	0.462	0.000	0.532	0.000
**Cut point**	A*. 120 kVp		B*. 80 kVp		C*. 80 kVp	
	**TPF (1-FNF)**	**FPF (1-TNF)**	**TPF (1-FNF)**	**FPF (1-TNF)**	**TPF (1-FNF)**	**FPF (1-TNF)**
0-1	0.608	0.326	0.655	0.132	0.749	0.178
1-2	0.561	0.194	0.655	0.078	0.737	0.109
2-3	0.433	0.085	0.596	0.000	0.673	0.000
3-4	0.298	0.000	0.509	0.000	0.591	0.000

**Figure 7:**
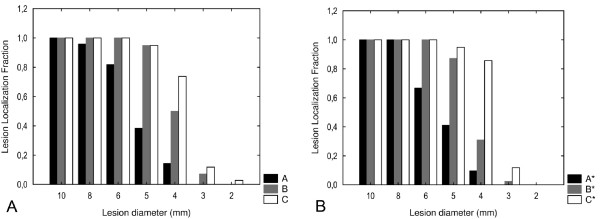
**(A-B) The reader-averaged LLF.** Interpreted with the fixed (**A**) and free-choice (**B**) widow setting. Presented for each lesion size (2 – 10 mm) and scanning protocol

**Figure 8:**
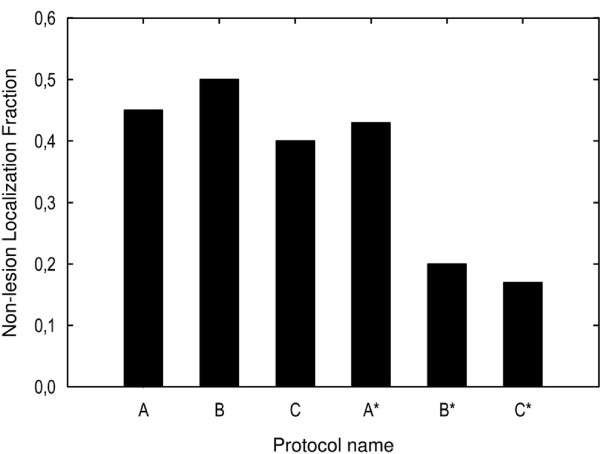
**The reader-averaged NLF for each scanning protocol interpreted with both free-choice and fixed window settings.** The smallest fractions (< 20%) of missed lesions was attained for the 80 kV protocol interpreted with the free-choice window setting

When the LLF and NLF were analyzed for each lesion size (Figures 7 and 8), smaller lesions were detected with 80 kVp than with 120 kVp. A major portion of the 5 mm lesions were detected at 80 kVp while only a small fraction of these were detected at 120 kVp. Also, a major proportion of the 4 mm lesions were detected at 80 kVp and 675 mA but not at 500 mA. The detection of lesions measuring between 2 and 3 mm was poor in all protocols.

## Discussion

The Catphan phantom was utilized in this study to represent the normal pancreatic parenchyma and was scanned by using various acquisition protocols, essentially varying the tube voltage between 120 kVp and 80 kVp. Computer-simulated, low attenuating lesions of various diameters, representing hypovascularised solid pancreatic tumors, were inserted into the ensuing phantom images. The rationale for using a low kilovoltage protocol was to achieve a higher attenuation difference between the tumor and the pancreatic parenchyma, in order to improve tumor conspicuity and delineation. Generally, lesions with attenuation nearly identical to that of the normal pancreatic parenchyma are difficult to visualize. By inserting 110 HU computer-simulated lesions, 20 HU less than that of the pancreatic phantom, we were able to mimic this clinical situation. When the tube voltage was decreased from 120 to 80 kVp, the mean photon energy decreased in parallel from 56.8 to 43.7 keV [18]. This lower value was closer to the K edge of iodine (33.2 keV) resulting in higher X-ray absorption and a significantly higher attenuation (67%) of the background (i.e. normal pancreatic tissue). Consequently, the attenuation difference between the digital lesions and the pancreatic background increases by 70% (from 20 HU to 34 HU).

The post-hoc analysis revealed significantly better lesion detection at 80 kVp than at 120 kVp, which means that smaller lesions and more of them are detected at 80 kVp. The main consideration for applying a lower tube voltage was the increase of image noise. In protocol C, we established the same image noise as in protocol A by using the maximum tube current possible with our 64-channel MDCT (675 mA). However, for lesions measuring ≥5 mm, the LLF at 80 kVp was not improved when the tube current was increased to 675 mA in order to establish the same image noise as in the 120 kVp protocol. In contrast, an increase of the tube current in the 80 kVp protocol (C) to achieve similar noise as in the 120 kVp protocol (A) improved the LLF for lesions with a diameter ≤4 mm.

The receiver operating characteristic (ROC) method has long been one of the standard methods in radiology to analyze and compare diagnostic accuracy [25].

The ROC method is very powerful because it estimates and reports all combinations of sensitivity and specificity that a diagnostic test is able to provide [26] and it is therefore used in this study. In the ROC paradigm, the observer is given a number of cases in some of which some kind of abnormality is present. The observer is asked to rate every case depending on how confident he or she is about whether there is an abnormality somewhere in that case.

The resulting 2 x 2 truth-response table defines correct decisions (true positives (TP) and true negatives (TN)) and incorrect decisions (false positives (FP) and false negatives (FN)) in comparison to a gold standard.

Pancreatic cancer incidence peaks between 60 and 80 years of age [27]. The risk of developing a radiation-induced cancer is markedly age-dependent. Given an estimated less than 5% 5-year survival rate, the risk to the patient associated with an increased radiation dose in order to achieve a technically optimal MDCT is negligible. We therefore believe that for patients with a high probability of pancreatic malignancy, the examination protocol should be tailored to achieve optimal tumor conspicuity. The radiation dose must, however, be taken into account for patients with hereditary or predisposing factors for pancreatic tumors (for example familiar syndromes and chronic pancreatitis) and subjects with the multiple endocrine neoplasia syndrome Type I (MEN-I) who undergo repeated screening controls.

When designing these examination protocols, it is therefore important to remember that the radiation dose will increase when the tube voltage is decreased because at the same time the tube current needs to be adjusted to maintain similar image noise. The reason for this is that the image noise is a function of the dose to the detector and not to the patient.

This study has some limitations. In daily clinical practice we do not consider the low kilovoltage protocols suitable for large patients (> 85 kg) because of the high image noise, despite the increase in radiation dose. Real tumors are not uniformly spherical in shape and are not always located in a perfectly homogenous background. Even though the vast majority of PDACs are hypoattenuated to pancreatic parenchyma, it should be noted that 11% of solid pancreatic malignancies are isoattenuating on CT [9]. In these cases the presence of secondary signs such as pancreatic and/or biliary duct dilatation can indicate the existence of a tumor. Also, the attenuation used as reference for pancreatic parenchyma (130HU) and the pancreatic cancers (110HU) were measured in a limited number of patients (n = 15). In the experimental situation a 20 HU attenuation difference between pancreas and tumor was thus assumed wheras in the clinical situation there is a variation in this respect. Furthermore, the simulated tumors were inserted into already reconstructed images, meaning that the lesions were not affected by the modular transfer function (MTF) of the system. In future studies, the lesions may instead be convolved with the point-spread function (PSF) before inserting them into the images in order to avoid this inconsistency.

Moreover, the study was designed as a free-response ROC (FROC), but the evaluation was performed as an ROC. Because two of the readers did not generate an appreciable amount of NLs for protocols B* and C*, the statistical analysis became less reliable. An ROC analysis was therefore performed, complemented with descriptive statistics, so that the information about the location and the number of lesions was not lost.

One of the readers did not use the same type of monitor (three mega pixel grey scale) as the other two. A one mega pixel color monitor was used instead. However, all monitors were calibrated according to DICOM part 14, and since CT images do not require high-resolution monitors, this difference was considered to be of small importance.

## Conclusion

In conclusion, by using this experimental model, we have shown that the low-kilovoltage, high-current MDCT improved the depiction of small, minimally hypodense, solid pancreatic lesions. However, further studies are needed to assess what the technique yields in the clinical setting.

## Competing interests

The authors declare that they have no competing interests.

## Authors' contributions

JH, LL, NA, NK, BL and AS participated in planning the study, executing the experiments, collecting and analysing the data and writing the manuscript. All the authors read and approved the final manuscript.

## Pre-publication history

The pre-publication history for this paper can be accessed here:

http://www.biomedcentral.com/1471-2342/12/20/prepub
